# Supplementary material for: Genomic Diversity of NDM-Producing Klebsiella Species from Brazil, 2013–2022
Source: Antibiotics (Basel). 2022 Oct 12;11(10):1395. doi: 10.3390/antibiotics11101395 (PMC9598336; doi:10.3390/antibiotics11101395)
Supplement: Supplementary file 1 [file antibiotics-11-01395-s001.zip › antibiotics-1943241-supplementary/Figure S1.pdf]

Figure S1. Dendrogram based on XbaI pulsed-field gel electrophoresis of NDM-producing *Klebsiella* spp. from Brazil. A total of 135 isolates are presented. The antimicrobial susceptibility profile is represented for each isolate, the black square indicates resistance; the gray one, susceptible, increased exposure, and the white, susceptible. Isolate number preceded by a filled circle was submitted to whole genome sequencing; the definitive identification based on genome analysis and the sequence type (ST) are indicated for those isolates. The new ST described in this study (ST6244, ST6245 [*K. pneumoniae*] and ST418 [*K. michiganensis*]) are indicated in red.

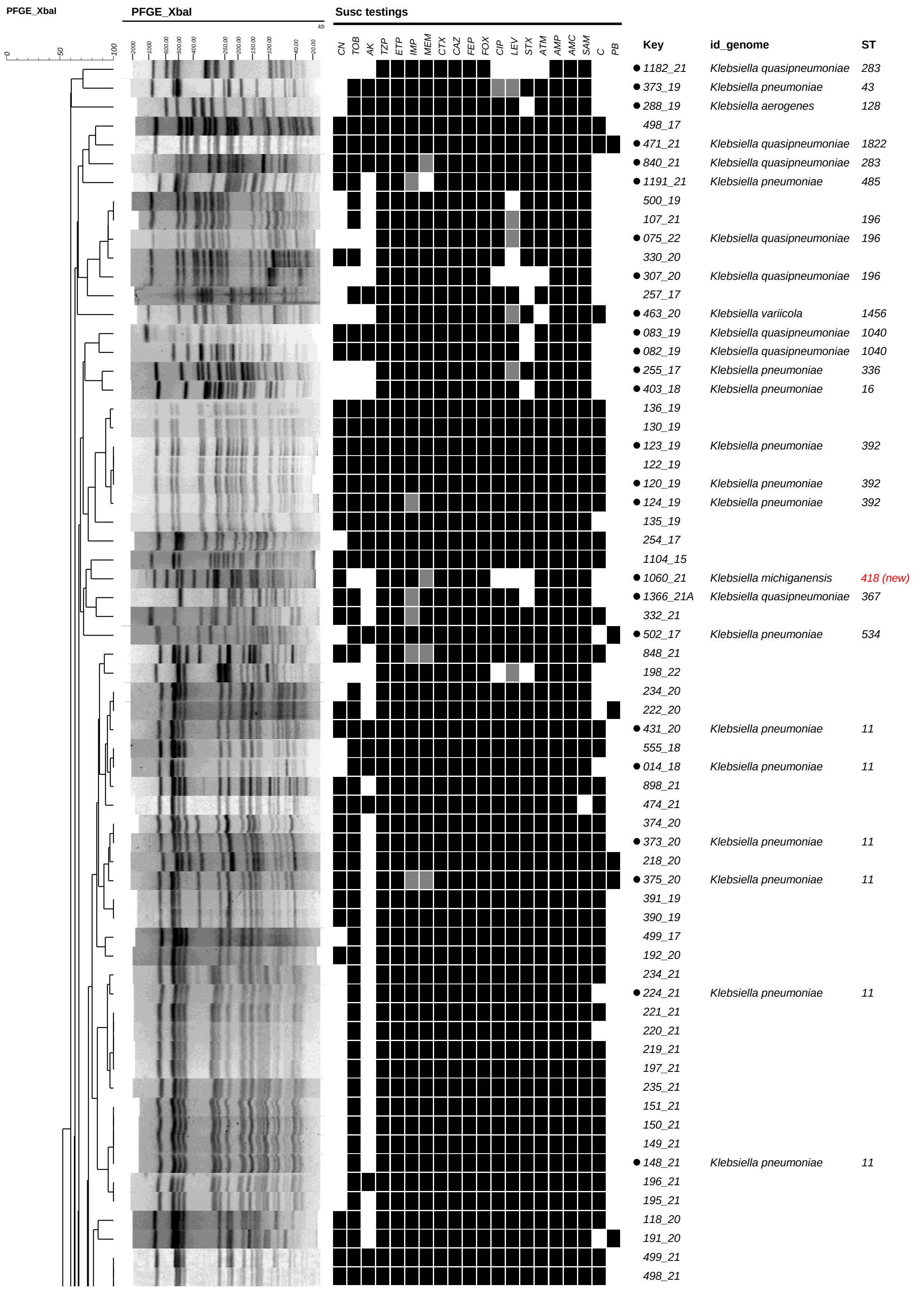

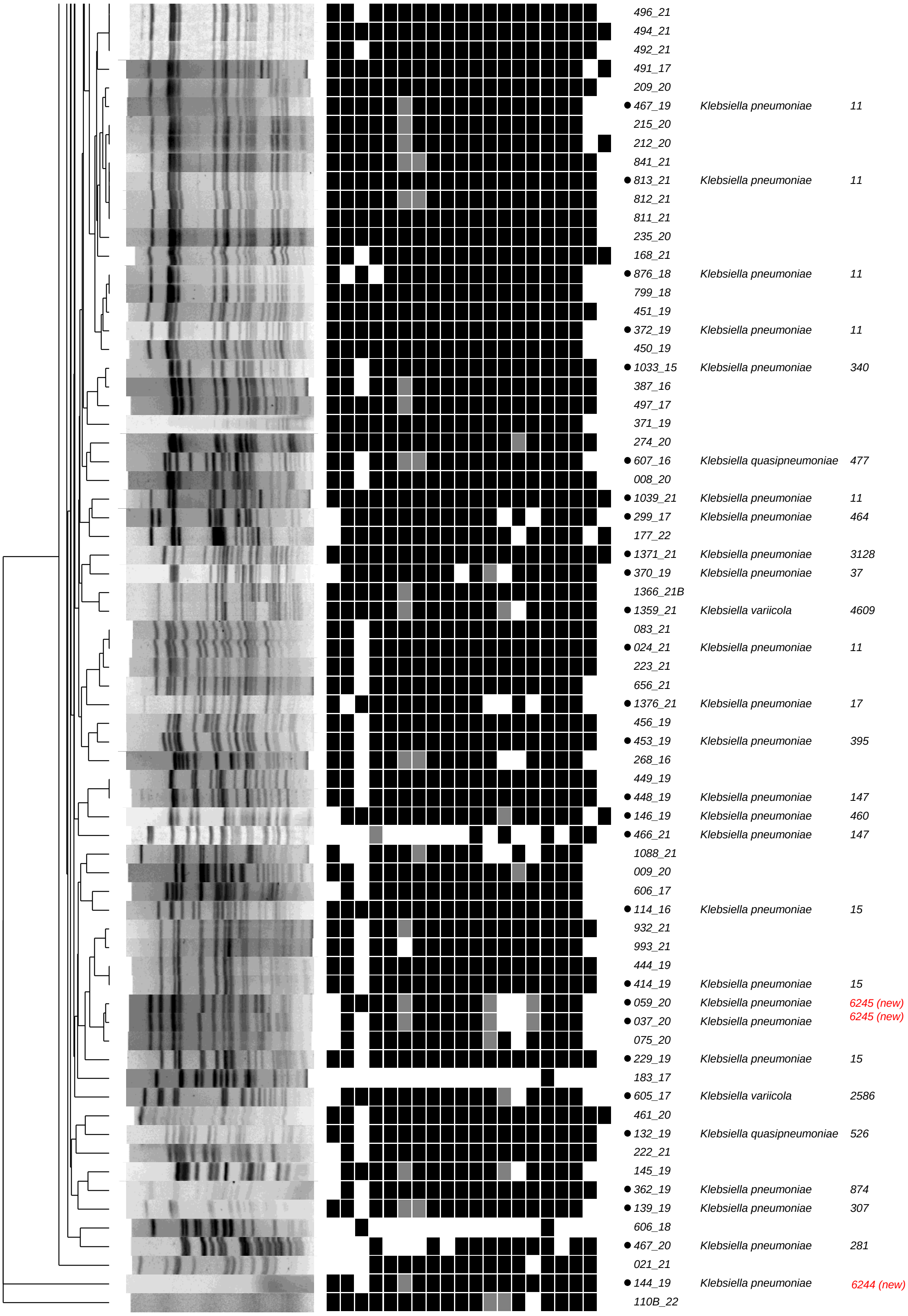

Legend

AK, amikacin; CN, gentamicin, TOB, tobramycin; CIP, ciprofloxacin; LEV, levofloxacin; SXT, trimethoprim-sulfamethoxazole; AMP, ampicillin; FEP, cefepime; CTX, cefotaxime; CAZ, ceftazidime; ETP, ertapenem; IMP, imipenem; MEM, meropenem; FOX, cefoxitin; AMC, amoxicillin-clavulanic acid; SAM, ampicillin-sulbactam; TZP, piperacillin-tazobactam; ATM, aztreonam; C, chloramphenicol; PB, polymyxin B.
