# Supplementary material for: Genomic Diversity of NDM-Producing Klebsiella Species from Brazil, 2013–2022
Source: Antibiotics (Basel). 2022 Oct 12;11(10):1395. doi: 10.3390/antibiotics11101395 (PMC9598336; doi:10.3390/antibiotics11101395)
Supplement: Supplementary file 1 [file antibiotics-11-01395-s001.zip › antibiotics-1943241-supplementary/Table S1.pdf]

Table S1. Distribution of NDM-producing *Klebsiella* from Brazil, according to State and Administrative Region.

Region/State	Total (n)	%	Sequenced (n)	%	Sequence types found *
Southeast	109	80.7	43	79.6	11 (12), 15 (3), 16, 17, 37, 43, 128, 147, 196 (3), 281, 283 (2), 336, 367, 395, 418, 464, 485, 534, 874, 1040 (2), 1456, 2586, 3128, 4609, 6245 (2)
Rio de Janeiro	1	0.7	1	1.9	464
São Paulo	107	79.3	41	75.9	11 (12), 15 (3), 16, 17, 37, 43, 128, 147, 196 (3), 281, 283 (2), 336, 367, 395, 418, 485, 534, 1040 (2), 1456, 2586, 3128, 4609, 6245 (2)
Minas Gerais	1	0.7	1	1.9	874
Midwest	1	0.7	0	0	-
Goiânia	1	0.7	0	0	-
Northeast	4	3.0	2	3.7	340, 477
Alagoas	1	0.7	1	1.9	477
Pernambuco	3	2.2	1	1.9	340
North	21	15.6	9	16.7	147, 307, 392 (3), 460, 526, 1822, 6244
Pará	8	5.9	2	3.7	147, 1822
Tocantins	13	9.6	7	13.0	307, 392 (3), 460, 526, 6244
Total	135	100	54	100	

***** In parenthesis are indicated the number of isolates, except when it was 1 (and it was omitted).
